# Image Processing Algorithms Analysis for Roadside Wild Animal Detection

**DOI:** 10.3390/s25185876

**Published:** 2025-09-19

**Authors:** Mindaugas Knyva, Darius Gailius, Šarūnas Kilius, Aistė Kukanauskaitė, Pranas Kuzas, Gintautas Balčiūnas, Asta Meškuotienė, Justina Dobilienė

**Affiliations:** 1 Department of Electronics Engineering, Kaunas University of Technology, Studentu Str. 50-457, 51368 Kaunas, Lithuania; sarunask@gmail.com (Š.K.); aiste.kukanauskaite11@gmail.com (A.K.); pranas.kuzas@ktu.lt (P.K.); 2 Metrology Institute, Kaunas University of Technology, Studentu Str. 50-454, 51368 Kaunas, Lithuania; darius.gailius@ktu.lt (D.G.); gintautas.balciunas@ktu.lt (G.B.); asta.meskuotiene@ktu.lt (A.M.); justina.dobiliene@ktu.lt (J.D.)

**Keywords:** wild animal detection, thermal imaging, image processing algorithms, motion detection, embedded systems, roadside surveillance

## Abstract

The study presents a comparative analysis of five distinct image processing methodologies for roadside wild animal detection using thermal imagery, aiming to identify an optimal approach for embedded system implementation to mitigate wildlife–vehicle collisions. The evaluated techniques included the following: bilateral filtering followed by thresholding and SIFT feature matching; Gaussian filtering combined with Canny edge detection and contour analysis; color quantization via the nearest average algorithm followed by contour identification; motion detection based on absolute inter-frame differencing, object dilation, thresholding, and contour comparison; and animal detection based on a YOLOv8n neural network. These algorithms were applied to sequential thermal images captured by a custom roadside surveillance system incorporating a thermal camera and a Raspberry Pi processing unit. Performance evaluation utilized a dataset of consecutive frames, assessing average execution time, sensitivity, specificity, and accuracy. The results revealed performance trade-offs: the motion detection method achieved the highest sensitivity (92.31%) and overall accuracy (87.50%), critical for minimizing missed detections, despite exhibiting the near lowest specificity (66.67%) and a moderate execution time (0.126 s) compared to the fastest bilateral filter approach (0.093 s) and the high-specificity Canny edge method (90.00%). Consequently, considering the paramount importance of detection reliability (sensitivity and accuracy) in this application, the motion-based methodology was selected for further development and implementation within the target embedded system framework. Subsequent testing on diverse datasets validated its general robustness while highlighting potential performance variations depending on dataset characteristics, particularly the duration of animal presence within the monitored frame.

## 1. Introduction

Animal migration is a fundamental biological phenomenon that plays a critical role in the life cycles of numerous species, influencing ecosystem dynamics, species interactions, and biodiversity patterns [1]. Monitoring animal migration is essential for understanding the spatial and temporal movements of species, which in turn provides insights into their behaviors, ecology, and responses to environmental changes. Such knowledge is indispensable for conserving biodiversity, managing wildlife populations, and mitigating the impacts of anthropogenic activities on natural habitats [2]. Furthermore, migration data are vital for informing environmental management strategies, particularly in the context of climate change, habitat fragmentation, and the conservation of migratory corridors [3].

To effectively monitor animal migration, a variety of methods have been developed, each with its own advantages and limitations. Satellite tracking has emerged as a powerful tool for studying long-distance migrations, particularly for large terrestrial and marine species. This method involves attaching satellite transmitters to animals, which transmit location data to orbiting satellites, enabling researchers to track movements across vast geographic scales [4]. GPS collars, a more precise alternative, provide high-resolution spatial and temporal data by recording and transmitting location information at regular intervals. These devices are particularly useful for studying the fine-scale movements of terrestrial mammals [5].

Radio telemetry, another widely used technique, relies on radio signals to track animals within a limited range. This method is cost-effective and suitable for studying smaller species or those inhabiting dense habitats where satellite or GPS signals may be obstructed [6]. Acoustic monitoring, on the other hand, is particularly effective for aquatic species, such as fish and marine mammals. By deploying hydrophones to detect and record acoustic signals emitted by tagged animals, researchers can monitor their movements and behaviors in underwater environments [7].

Each of these methods contributes uniquely to the study of animal migration, offering complementary insights into the spatial ecology and behaviors of migratory species. However, the choice of monitoring techniques depends on factors such as the species’ size, habitat, and migratory range, as well as the specific research objectives [8]. Despite the advancements in tracking technologies, challenges remain, including the high costs of equipment, the need for miniaturization for smaller species, and the ethical considerations of tagging and handling animals [9].

Also, animal migration across roads poses significant risks to both wildlife and human safety, with far-reaching ecological and socio-economic consequences. As migratory species traverse roadways, they face heightened mortality rates due to vehicle collisions, while drivers and passengers are exposed to potential accidents and injuries. In Europe, the expansion of road networks has increasingly fragmented habitats, disrupting migratory pathways and exacerbating the frequency of wildlife–vehicle collisions (WVCs). For instance, in Europe, it is estimated that over 500,000 WVCs occur annually, resulting in substantial economic costs and ecological impacts [10,11].

The risks to human safety are equally significant. In the European Union, WVCs result in approximately 30,000 human injuries and 300 fatalities annually, with economic costs exceeding EUR 1 billion per year due to medical expenses, vehicle repairs, and emergency response [10]. A more recent study in Great Britain showed that there are, on average, 42,500 to 74,000 accidents/year, 550 human injuries, and 12 human fatalities per year, with a cost of around EUR 25 million per year [12]. Large mammals, such as deer and wild boars, are particularly hazardous due to their size and the potential for severe vehicle damage. These incidents are most common during peak migration seasons, such as autumn and spring, and in regions where roads intersect critical migratory corridors, such as forested areas and river valleys. Addressing these challenges requires a combination of infrastructure development, public education, and policy interventions. By integrating scientific research with practical solutions, it is possible to mitigate the impacts of roads on migratory species while enhancing safety for drivers and passengers.

To reduce the number of animal–vehicle collisions, the strategies used can be divided into three main types: structural, behavioral, and technological. Structural methods involve physical modifications to infrastructure, such as fencing and wildlife crossing structures, designed to block or redirect animal movement across roads. These systems effectively prevent animals from entering highway zones, especially when fences are paired with crossing structures like underpasses, enhancing permeability and habitat connectivity. However, they require regular maintenance and monitoring due to potential issues like poor design, substandard materials, snow accumulation, fallen trees, or vandalism, suggesting a potentially high cost for upkeep and quality construction [13]. Behavioral methods leverage animal responses to sensory or environmental cues, including acoustic deterrents and vegetation management, to discourage road access. Technological methods employ advanced systems, such as dynamic warning systems and artificial intelligence, to detect wildlife presence and enhance driver awareness.

Responding to the limitations and costs of traditional fence-crossing systems, Roadside Animal Detection Systems (RADSs) emerged as an alternative mitigation strategy in the 1990s [14]. First tested in Switzerland [15], RADSs aim to warn drivers of nearby wildlife, rather than physically preventing animals from accessing the road. These systems detect animals using roadside sensors (e.g., infrared, thermal, or motion-activated), which then trigger flashing warning signs for drivers. According to the report [16], most RADS technology relies on either area cover sensors, which detect animals anywhere within their coverage, or break-the-beam sensors, which register animals as they interrupt a signal beam upon entering or leaving a roadway.

Break-the-beam systems operate by transmitting a continuous signal—such as infrared, laser, or microwave—between a paired transmitter and receiver positioned across a roadway or along its perimeter. The interruption or attenuation of this signal, caused by an object obstructing the beam, triggers an alert mechanism. In the context of wildlife detection, the system activates when an animal crosses the beam’s path, enabling timely warnings to motorists or initiating deterrent measures to prevent animals from entering the roadway. Break-the-beam systems are valued for their straightforward design and prompt response capabilities. Their effectiveness is particularly notable in scenarios where wildlife crossings are concentrated at specific points, such as known animal trails or designated crossing structures. By installing these systems at strategic locations, they can effectively monitor and detect large animals approaching the roadway, subsequently triggering warning signals to alert drivers. This proactive approach has been implemented in various regions to reduce WVCs, contributing to enhanced road safety and wildlife conservation [17].

Huijser and Hayden [18] evaluated the reliability of an animal detection system designed to reduce wildlife–vehicle collisions, focusing on a microwave break-the-beam system manufactured by ICx Radar Systems. The system, tested in a controlled environment in Montana, uses microwave radio signals to detect large animals such as deer, elk, and moose, triggering warning signs for drivers when animals are near the road. The study employed domesticated animals (horses, llamas, and sheep) as models for wildlife to assess the system’s performance under various conditions. The results showed that the system had a low rate of false positives and false negatives, with 97% of animal intrusions detected. However, the system experienced significant downtime (40%) during a snowstorm, suggesting that adverse weather conditions, such as snow and ice buildup on sensors, can impair functionality. The findings suggest that while technology is effective in reducing wildlife–vehicle collisions, its reliability in harsh weather remains a concern.

Knyva et al. [19] propose an IoT-based sensor network for detecting wild animals near roads to mitigate wildlife–vehicle collisions. The system comprises detection–signaling nodes (DSNs) equipped with passive infrared (PIR) sensors, LoRa wireless communication, and solar-powered batteries. Installed on roadside marker posts at 50 m intervals, DSNs detect animal movement and activate visual warning signals for drivers. In the research setup, a gateway collects sensor data, backed up by a thermal camera image dataset. Field tests with later data processing showed that the system effectively identifies wildlife presence. Some challenges, such as optimizing sensor placement and improving resistance to environmental interference, still prevail.

With advancements in technology, modern animal-monitoring systems increasingly rely on camera-based detection, incorporating a variety of methods, models, digital image processing techniques, as well as machine learning and deep learning algorithms to enhance image analysis and recognition.

Nandutu et al. [20] review intelligent systems that utilize sensors and machine learning to mitigate wildlife–vehicle collisions (WVCs). The study highlights the increasing global concern over WVCs, which result in human and animal fatalities and significant economic costs. The review suggests that integrating state-of-the-art machine learning models, such as convolutional neural networks (CNNs) and ensemble learning techniques, can significantly improve detection accuracy. Additionally, the authors identify key challenges, including the need for better dataset availability. The challenges of animal species recognition and the detection of small animals are addressed.

Bakana et al. [21] introduce WildARe-YOLO, a lightweight and efficient deep learning model for wild animal recognition, designed to address computational constraints in real-time wildlife monitoring. The model is an optimized version of YOLOv5s, incorporating Mobile Bottleneck Block modules and an improved StemBlock to reduce floating-point operations (FLOPs) and parameter count. It employs Focal-EIoU loss for precise bounding box predictions and a BiFPN-based neck for improved feature extraction. While it demonstrates strong performance, challenges such as recognition in complex environments and real-time deployment under varying atmospheric conditions remain topical areas for future research.

Road scene understanding and object recognition are also essential for autonomous driving, enabling reliable perception of complex environments. To address geographic limitations of existing datasets, Zunair et al. [22] introduce RSUD20K, a large-scale dataset comprising 20,334 images with diverse road scenes from Bangladesh. The authors benchmark state-of-the-art detectors, including YOLOv6, YOLOv8, DETR, and RTMDET, demonstrating the challenges of object recognition in dense, cluttered conditions, including rainy and night scenes. The results show that YOLOv6 and YOLOv8 achieve the best performance (reaching 73.7 mAP and 71.8 mAP, respectively), demonstrating superior accuracy in unique and complex contexts. The method is applicable for the analysis of images captured from the driver’s perspective.

The development of advanced video-based monitoring systems has introduced new challenges, particularly in rural areas where infrastructure is limited. These systems, which rely on high computational power for tasks like image processing and object recognition, often require significant data transmission rates to handle video streams effectively. However, rural environments face unique obstacles, such as long distances between nodes and limited energy resources, making traditional urban-focused technologies like Zigbee, Bluetooth, or Wi-Fi unsuitable. These limitations hinder the deployment of wireless multimedia sensor networks (WMSNs) in rural settings, where applications such as agricultural monitoring or wildlife observation could greatly benefit from real-time video streaming. Addressing these challenges is crucial for enabling efficient and reliable data transmission in remote areas. Zaragoza-Esquerdo et al. [23] explore the feasibility of using LoRa (Long Range) technology for video streaming in rural Wireless Multimedia Sensor Networks (WMSNs) for smart agriculture. The study proposes a network architecture with sensor nodes, multimedia nodes (equipped with cameras), and aggregator nodes to monitor agricultural fields. The researchers tested video transmission between an Arduino-based emitter node and an ESP32-based receiver node using LoRa UART SX1278 modules. The study highlights that LoRa, despite its bandwidth limitations, can support low-resolution video streaming in rural areas, making it suitable for applications like intruder detection or crop monitoring. Future work aims to extend transmission distances and evaluate the impact on other sensor nodes within the network. This research demonstrates the potential of LoRa for cost-effective, long-range video streaming in agricultural monitoring systems.

The methods of wild animal detection based on image capturing using thermal imaging or night vision solutions offer more flexibility, allowing the application of image processing algorithms. In the development stage, the image acquisition system provides a background context for better comprehension of the beam barrier crossing and thus enables them to address false negatives and their causes. Expert preview analysis of the image database is sufficient for initial estimation of the scene and gives base estimates about species and their behavior at the investigated scene. The embedded image processing solution, however, in most cases, works in limited electrical power resource conditions, and the processing algorithms require optimization in terms of calculation resources with maximum available reliability of object detection.

In this study, we present a comparative analysis of four distinct image processing methodologies for detecting wild animals in an experimentally collected roadside thermal imagery dataset, specifically targeting suitability for embedded system deployment. Our research evaluates algorithms based on bilateral filtering, Canny edge detection, color quantization, and inter-frame motion detection using data captured by a custom thermal surveillance setup. The study compares traditional image processing methods for their suitability in resource-constrained embedded systems with a lightweight framework. A key consideration was that wild animals, such as deer and wild boars, predominantly migrate during nighttime hours. While ML algorithms often necessitate high-quality visual data for robust feature extraction, conventional cameras for nighttime detection would struggle with visibility issues and require additional limited-range IR illumination, which may be costly. Therefore, thermal cameras were specifically chosen as they reliably capture heat signatures and detect animal movement regardless of lighting or environmental conditions. This approach allows for effective identification of animal presence within the monitored area, enabling prompt warnings to drivers of potential danger.

## 2. Roadside Video Surveillance System

**Figure 1:**
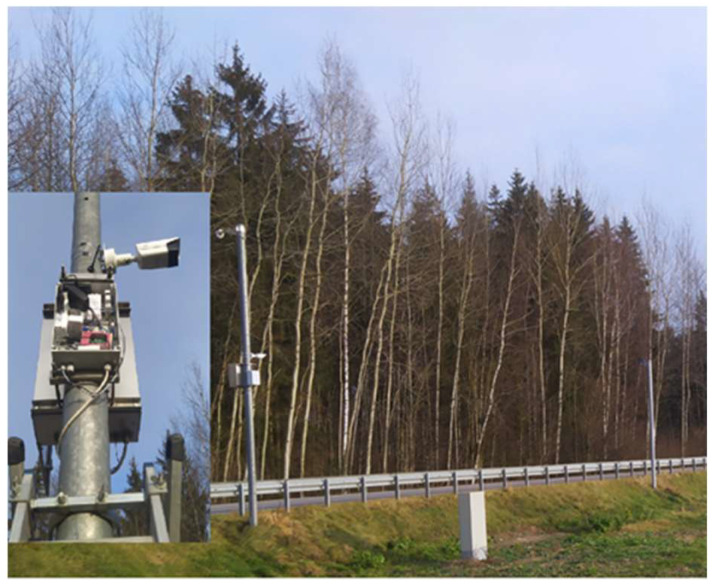
Data collection system.

The proposed video surveillance system is designed by the authors at Kaunas University of Technology for detecting animals near roadways. The system utilizes a video camera to monitor roadside areas and capture footage in both color and infrared (IR) modes. A video camera continuously records the roadside area, capturing images in both visible and infrared spectra. A real view of the data collection system on the road Kaunas–Šakiai is presented in Figure 1.

The system consists of two cameras facing towards the road with thermovision function, a 4G modem/router, and a Raspberry Pi 4 microcomputer (Raspberry Pi, Cambridge, UK) used for camera control and data collection. When a heat-emitting body approaches the road, the camera reacts to the activation of the sensors.

Using the cameras, data is also collected in the form of photos and stored in a database. DS-2TD2617-6/PA thermal imagers (Hikvision, Hangzhou, China) are used; the resolution of the photos obtained by them is 320 × 240 pixels [24]. A thermal imaging camera was utilized in the system due to its ability to detect and capture heat emitted by living bodies. In contrast, conventional cameras that rely on reflected light may struggle to distinguish animals in the captured images, particularly when the animals blend into the color patterns of the surrounding environment or when visibility is reduced by adverse weather conditions. The use of a thermal camera effectively eliminates this limitation: in thermographic images, animals remain clearly distinguishable from grassy backgrounds, as the ground emits significantly less heat compared to the body of a living animal.

**Figure 2:**
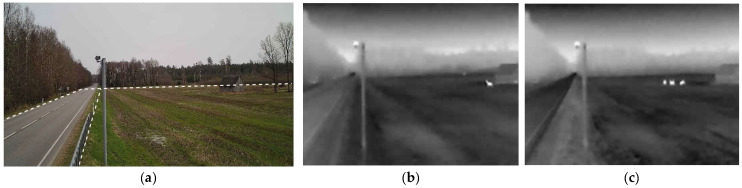
Environmental context and animal detection: (**a**) A colored photograph of the study area; (**b**,**c**) animals captured using an IR camera.

Figure 2 presents the environmental context and animal detection using infrared (IR) cameras. Figure 2a displays a colored photograph of the study area, while Figure 2b,c show animals detected using IR camera technology. The IR images highlight the animals’ thermal signatures, enabling clear identification even in low-light conditions.

**Figure 3:**
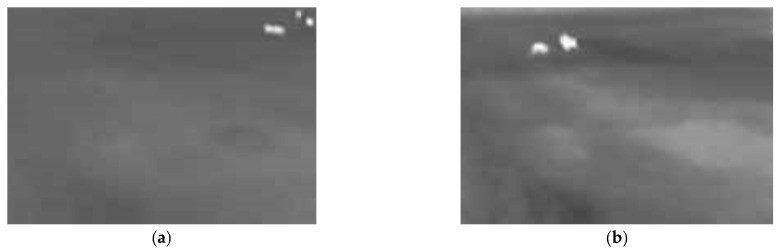
Examples of cropped photos with visible animals: (**a**,**b**) animals captured using IR camera.

Captured images undergo preprocessing to crop the relevant region of interest (ROI), focusing solely on the roadside grassland area. This step is essential to eliminate unnecessary parts of the image, such as the road and the sky, which may introduce noise and interfere with accurate animal detection. Several main areas of the image were selected by separating the ROIs: a horizontal line separating the forest from the meadow, a vertical line separating the road from the meadow, and a diagonal line separating the road from the forest on the opposite side of the road. The examples of cropped photos are shown in Figure 3. Cropped images like this were used in the experiment to design the algorithms being analyzed.

Without this cropping step, image analysis could be affected by reflections on the road surface and thermal signatures from non-animal objects, leading to false detections. The system’s preprocessing stage minimizes these interferences, improving the reliability of animal identification.

Over the course of a single day, approximately 8000 images were collected. Over four seasons, the database containing over 800,000 images was formed. From this pool, the thermal images were selected from various days and seasons, resulting in the creation of five distinct datasets for experimental analysis. For each image, the a priori knowledge of whether an animal was present or if it only contained seasonal obstacles was assigned, allowing for precise evaluation of the methodology’s detection capabilities.

## 3. Methods for Animal Detection in the Thermovision Pictures

This section details the specific image processing algorithms developed and evaluated for detecting animals within the thermal images captured by the roadside surveillance system. Five distinct methodologies are presented: three approaches analyze individual image frames using bilateral filtering, Canny edge detection, and color quantization techniques; the fourth method leverages motion detection by comparing consecutive frames; and the fifth methodology is based on a YOLOv8n neural network. The summarized sequence of applied actions consists of the following main steps:

- Image input;
- Image cropping;
- Image processing;
- Result output.

The exact application process of each specific algorithm, from initial filtering to final object identification, is described in the subsequent subsections

### 3.1. Animals’ Detection Using Bilateral Filter and Thresholding

**Figure 4:**
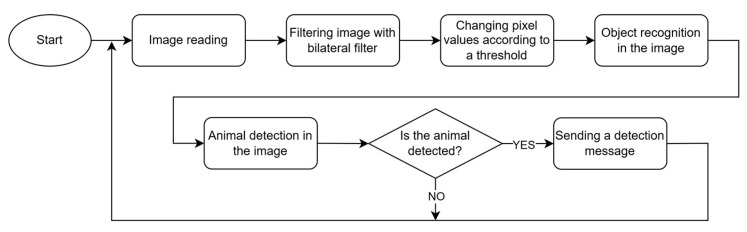
Flowchart of animals’ detection using bilateral filter and thresholding.

The execution algorithm of the first method for detecting animals is presented in Figure 4.

The execution algorithm defines a sequential image processing pipeline for animal detection. It starts with image acquisition, followed by essential preprocessing steps. Initially, a bilateral filter is applied to the input image, aiming to reduce noise while preserving significant edge features crucial for subsequent analysis. Following filtration, pixel values are adjusted via thresholding, effectively segmenting the image based on intensity levels to isolate potential objects from the background. The subsequent object recognition phase involves identifying distinct entities within the processed image, likely utilizing contour detection techniques. A dedicated animal detection module then analyzes these recognized objects, comparing their characteristics or contours against predefined animal models. Based on this classification step, a conditional branch determines the system’s response: if an animal is successfully detected, a corresponding notification message is dispatched; otherwise, the system proceeds to await the next image frame for analysis, implicitly restarting the detection cycle.

#### 3.1.1. Bilateral Filter

In the first method, the image was filtered using a bilateral filter, designed to remove Gaussian noise in images [25]. The bilateral filter is a widely used non-linear filtering technique in image processing, known for its ability to smooth images while preserving edges and fine details. Unlike traditional linear filters, such as Gaussian blur, the bilateral filter incorporates both spatial and intensity information, making it particularly effective for noise reduction and edge preservation tasks [26].

The bilateral filter smooths an image while preserving edges by combining domain (spatial) and range (intensity) filtering. The filtered intensity at a pixel is given by

(1) $$I_{filtered}\left(x\right)=\frac{1}{W_{p}}\sum_{x_{i}\epsilon Ω}I(x_{i})f_{r}(\left|I(x_{i}-I(x)\right|)f_{x}\left(\left\|x_{i}-x\right\|\right),$$

where ***I***(***x***) is the input image intensity at pixel ***x***; *Ω* is the neighborhood of pixel ***x***, *f_s_*(||***x**_i_* − ***x***||) is the spatial kernel, typically a Gaussian function that assigns weights based on Euclidean distance; *f_r_*(|***I***(***x**_i_*) − ***I***(***x***)|) is the range kernel, also typically a Gaussian function, that assigns weights based on intensity similarity; and *W_p_* = *Σ_xi__ϵ__Ω_f_r_*(|***I***(***x**_i_*) − ***I***(***x***)|) *f_s_*(||***x**_i_* − ***x***||) is a normalization factor ensuring the filter conserves image brightness.

The spatial kernel ensures that closer pixels contribute more to the filtering process, while the range kernel prevents excessive blurring across edges by reducing the influence of pixels with significantly different intensity values.

In practice, the spatial kernel *f_s_* and the range kernel *f_r_* are chosen as Gaussian functions:

(2) $$f_{s}\left(\left\|x_{i}-x\right\|\right)=\exp\left(-\frac{{\left\|x_{i}-x\right\|}^{2}}{2\sigma_{s}^{2}}\right),$$

(3) $$f_{r}\left(\left|I(x_{i}-I(x)\right|\right)=\exp\left(-\frac{{\left|I\left(x_{i}\right)-I(x)\right|}^{2}}{2\sigma_{r}^{2}}\right),$$

where *σ_s_* and *σ_r_* control the spatial and intensity similarities, respectively.

It is worth noting that the edge sharpness in the filtered image is preserved because the weights depend not only on the Euclidean distance between pixels but also on radiometric differences (e.g., range differences such as color intensity, depth distance, etc.) [27]. For the implementation of the method, a window size of *Ω* = 5 × 5 pixels was selected; this window size was chosen to enable the filter to operate in a real-time system. Additionally, the distance for determining pixel neighborhoods when calculating weight coefficients was set to 9, and the smoothing parameters *σ_s_* and *σ_r_* were chosen to be equal, with their values set to 75.

#### 3.1.2. Selection of an Appropriate Threshold

Thresholding is performed to convert a grayscale image, where pixel values range from 0 to 255, into a binary image with pixel values limited to either black (0) or white (1) [28]. This transformation is necessary for the next methodological step described in the following section. The binary image is obtained using the following mathematical expression:

(4) $$I_{proc}\left(x,y\right)=\left\{\begin{array}{c} I_{max},\;\;\;\;\;\;\;\;\;\;\;if\;I_{org}\left(x,y\right)>threshold\\ 0,\;\;\;\;\;\;\;\;\;\;\;\;\;\;\;\;\;if\;I_{orig}(x,y)\leq threshold\;\; \end{array}\right.$$

where ***I****_proc_*(***x***,***y***) represents the pixel coordinates of the processed image after thresholding, ***I****_orig_*(***x***,***y***) represents the pixel coordinates of the original image, *I_max_* is the predefined maximum allowable pixel intensity value, and *threshold* is the selected threshold value that determines the pixel classification. For the implementation of the method, the chosen threshold value is set to 180.

#### 3.1.3. Finding the Contours of Objects

The object contour detection algorithm requires a binary image. Since calculations are performed with only two intensity values, it can be said that the pair of two pixels where the intensity of the pixel changes from 0 to 1 contains a contour edge [29]. In this way, all such changes in pixel intensity can be found, and the pixels that make them up, separated by one pixel, can be assigned to specific groups. A separate group of changes in the intensity of captured pixels is called an object contour.

When finding contours of objects, it was chosen to compress horizontal, vertical, and diagonal segments, keeping only the necessary contour shape to define pixel coordinates. Objects found in the photo that intersect with the edge of the photo are also programmatically rejected, thus minimizing the detection of inappropriate objects.

#### 3.1.4. Finding Contour Matches for Animal Objects

After finding the contours of objects, distinctive features are selected within the boundaries of the contours, in other words, features that describe the uniqueness of the object. From the photos obtained by the camera installed in the system, pre-selected pictures with animals and pictures of animal objects are cropped and added to the dataset. Using this database and knowing that there is indeed an animal in the photo in the database, distinctive features are also selected for its subject in the image. These features are selected from real-time photos and features obtained from photos of animal objects, and their definitions are compared with each other. The selection of features in computer vision is called feature detection, and the description of features so that they can be found not only in the original but also in other photos is called feature description. Algorithms for selecting and defining unique features are implemented in the OpenCV library. For the implementation of this stage of the methodology, the SIFT algorithm was chosen, and features in different photos were compared using the Brute Force matching algorithm [30].

SIFT, the scale invariant feature transform (scale invariant feature transform) algorithm, consists of four steps. First, using the Laplacian of Gaussian at different scaling parameter values and performing the Difference of Gaussians approximation, potential key features are found at different scales and in different locations in space. Potential feature points are then filtered by removing low-contrast points based on the intensity of the extreme point and by removing edge points of objects. In the third step, the assignment of orientation to the points of the main features is carried out: compounds of points of the same scale and in the same place are created, which differ in their direction and angle of rotation. Finally, the neighboring pixel values of the key point are marked to define the feature, and orientation diagrams are created from the decomposed matrix of neighboring pixel values and saved as vectors.

The direct matching algorithm compares the feature definitions found in both images using the distance metric and finds feature points that potentially correspond to similar locations in the images based on the shortest distance between them.

Animal detection in objects found in photos finds key features and their definitions for both objects and cropped animal photos. These features are then compared, and if the features found in the contours of the object match the features of an animal in the dataset, the photo is said to contain an animal.

#### 3.1.5. Results

**Figure 5:**
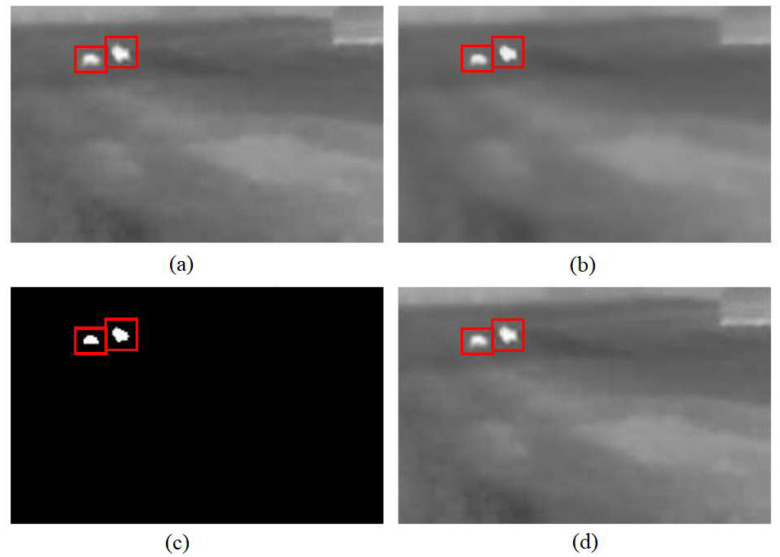
The images processed using a bilateral filter and thresholding. (**a**) Original image captured by the thermal camera. (**b**) The results after applying a bilateral filter to reduce noise. (**c**) Binary images obtained through thresholding to isolate high-temperature regions, potentially indicating animal presence. (**d**) The final result after contour detection to identify the shapes and positions of animals within the frame.

Figure 5 presents an unaltered image with animals and illustrations of the first steps of the methodology: filtration with a bilateral filter, thresholding, and recognition of animal contours. As can be seen in Figure 5, filtering with a bilateral filter removes the noise existing in the image, smooths it, but the edges of the objects visible in the form of white color remain as sharp. A suitable threshold allows the object recognition algorithm to present white blobs representing objects on a black background. Two animal objects were detected in the sample photo; in the original photos and the photos illustrating the steps of the methodology, the animal objects are highlighted by red rectangles.

### 3.2. Animals’ Detection Method Based on the Canny Edge Finding Algorithm

**Figure 6:**
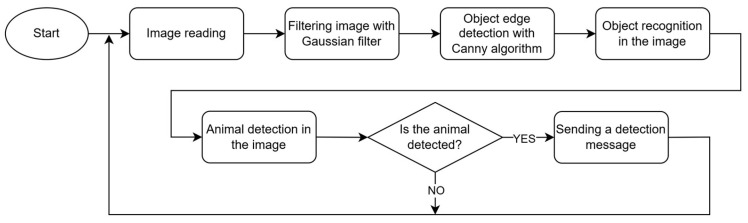
Flowchart of animals’ detection using Canny algorithm.

The algorithm of the second method for detecting animals is presented in Figure 6.

The initial preprocessing stage uses a Gaussian filter to gently blur the image, which helps to reduce visual noise like speckles or graininess, making the next steps more reliable. Following the blurring, the Canny algorithm is employed to precisely identify the edges or outlines of objects within the image. Based on these detected edges, object recognition is performed, typically involving contour analysis to delineate potential objects within the scene. The process finishes with a conditional decision: if an animal is positively identified, a detection message is sent; otherwise, the system cycles to await and process the next available image frame, concluding the current iteration without notification.

#### 3.2.1. Blurring with a Gaussian Filter

Before applying the Canny edge detection algorithm to extract object boundaries, it is necessary to minimize the noise in the photo. To achieve this, it was chosen to filter the image with a Gaussian filter [31]. A filter is applied to the image, described by the following equation:

(5) $$K\left(x,y\right)=\;\frac{1}{2\pi \sigma^{2}}exp\left(-\frac{{\left(i-(k+1)\right)}^{2}+{\left(j-(k+1)\right)}^{2}}{2\sigma^{2}}\right);\;\;\;\;\;1\leq x,y\leq \left(2k+1\right),$$

where ***K*** is the filter kernel mask, ***x*** and ***y*** are the coordinates of a specific pixel to be filtered, *σ* is the blurring parameter, and (2*k* + 1) is the filter mask size and number of rows and columns.

An example filter, when the parameter k is equal to 1, so the number of rows and columns of the filter is equal to 3, is shown below:

(6) $$I_{filtered}=\frac{1}{28}\left[\begin{array}{ccc} 2 & 4 & 2\\ 4 & 8 & 4\\ 2 & 4 & 2 \end{array}\right]*I_{original},$$

where ***I****_filtered_* is the filtered picture, and ***I****_original_* is the original picture in the input. The matrix of coefficients determines the weighted influence of neighboring pixels when calculating the intensity value of the central pixel. The asterisk symbol “*” in the formula describing the operation of the filter defines the convolution operation: filtering starts from the top left corner by placing the center filter pixel to the pixel closest to the top left corner of the image, so that the filter mask touches the edge and corner filter pixels. After calculating the value of the central pixel, the filter is shifted by one pixel step to the left, when reaching the end of the line, to the starting pixel of the next line; the calculation operation is performed on the former pixel values, and the new pixel intensity values are stored in memory.

Using this filter, object edge sharpness is lost, but the image is blurred, thus removing noise and smoothing out changes between the background and irrelevant objects that should not be detected, such as the reflections of scores [32]. It is not necessary to ensure the sharpness of object edges before using the Canny algorithm. The filter mask size chosen to implement the method is 3 × 3 pixels.

#### 3.2.2. Canny Edge Detection Algorithm

Since the edge of an object in an image can be rotated in different directions, the Canny algorithm typically uses four filters to detect horizontal, vertical, and diagonal edges in a blurred image [33]. During the edge detection operation (the Sobel edge detection operator is chosen to be used in this methodology), two convolution masks are applied in the x and y directions, as seen below:

(7) $$G_{x}=\left[\begin{array}{ccc} -1 & 0 & +1\\ -2 & 0 & +2\\ -1 & 0 & +1 \end{array}\right],\;\;G_{y}=\left[\begin{array}{ccc} -1 & -2 & -1\\ 0 & 0 & 0\\ +1 & +2 & +1 \end{array}\right],$$

Using masks, a filtering procedure is performed on the image based on the principle of convolution. Masks drawn on a new central pixel allow the gradient and direction of the object’s edge to be calculated:

(8) $$G=\sqrt{G_{x}^{2}+G_{y}^{2}},\;\;\;\;\;\;\;\;\theta =arctan\left(\frac{G_{y}}{G_{x}}\right),$$

where ***G*** is the strength of the edge gradient, *θ* is the direction of the edge gradient, and ***G****_x_* and ***G****_y_* are the first-order derivatives of the pixel group intensities filtered by masks acting in the corresponding x and y directions. Edge gradient direction is assigned to one of four possible values according to its value: 0°, 45°, 90°, and 135°.

Next, the edge thinning technique is applied [34]. For each pixel in the gradient image, the edge strength between the current pixel and the pixels in the positive and negative directions of the gradient is compared. If the edge strength for the current pixel is the highest compared to other pixels under the same mask and considered part of the gradient in the same direction, this pixel will be considered part of the edge of the object. Otherwise, it will be rejected.

After the edge thinning operation, pixels with strong and weak gradient values are double-thresholded as part of the object’s edge [33]. Since some pixels may be considered as part of an edge but are random in the calculations due to color range or noise, it is necessary to consider only those pixels that have a high gradient value as edges. This is performed by selecting high and low threshold values. If the pixel value is higher than the high threshold value, it is considered a strong edge pixel. Otherwise, if the pixel value is between the two threshold values, this pixel is considered a weak edge pixel; if the pixel value is lower than the lower threshold value, the pixel is rejected. Finally, when analyzing weak pixels, those in contact with strong pixels are accepted; other weak pixels are discarded, and strong ones are accepted as object edges.

The output of the Canny algorithm is a binary image in which the highest-intensity white pixels correspond to the edges of objects, and the lowest-intensity black pixels correspond to the background and fill of objects. The binary image is further processed by the object contour detection algorithm described in Section 3.1.3, and it is searched for animal objects using the technique described in Section 3.1.4.

#### 3.2.3. Results

**Figure 7:**
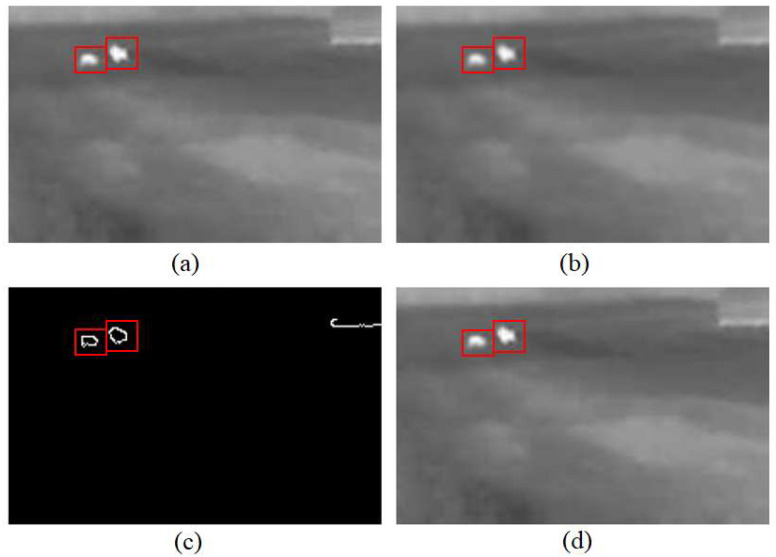
The image processed using the Canny edge finding algorithm. (**a**) The original image captured with a thermal camera. (**b**) The image after applying Gaussian filtering to reduce noise and preserve object boundaries. (**c**) Detected edges using the Canny algorithm, highlighting potential object contours. (**d**) The final detected animal contours superimposed on the original image. Red bounding boxes indicate the identified animal objects throughout the processing pipeline.

Figure 7 shows the unaltered photo with animals and illustrations of the steps of implementing the second methodology: filtering with a Gaussian filter, determining the contours of objects with the Canny algorithm, and recognizing the contours of animals. After filtering with a Gaussian filter, the contours of the white objects in the top right photo become fainter than in the top left photo. A photo processed by the Canny algorithm provides information about the objects found in the illustration in the form of white outlines. As can be seen, not only were the contours of animal objects highlighted, but the outline of the bottom of the structure was also visible from them. Using part of the animal object detection algorithm, two animals were detected; both in the original and in the photos illustrating the steps of the methodology, the animal objects are highlighted by red rectangles.

### 3.3. Animals’ Detection Method Based on Color Quantization by the Nearest Average Algorithm

#### 3.3.1. A Method Description

Color quantization is an image editing technique that allows for a reduction in the number of colors used to represent an image [35]. This method was chosen to preprocess the image to reduce the number of gray tones in the photo. In this way, the color gamut of the image would be grouped into a specified number of color groups to distinguish objects in the images captured by the camera, which would be radiating heat and yet stand out from the general background.

To perform the color quantization procedure, it was chosen to use the nearest average algorithm [36]. By applying this algorithm, the intensity values of the input data—image pixels—are grouped into a specified number of clusters depending on the size of the value.

First, the first step of the algorithm is carried out; a specified number of central group points and the pixel intensity values are randomly selected. The Euclidean distance from each point to the central points is then calculated, and the pixels are accordingly marked as belonging to the group with the smallest distance to the intensity of the central pixel, according to the following formula:

(9) $$G_{i}^{\left(t\right)}=\left\{x_{p}:{\left\|x_{p}-m_{i}^{\left(t\right)}\right\|}^{2}\leq {\left\|x_{p}-m_{j}^{\left(t\right)}\right\|}^{2}\forall j,\;\;\;1\leq j\leq k\right\},$$

where *k* is the number of groups, ***m****_i_* and ***m****_j_* are, respectively, the center point of the newly assigned and current groups assigned to the pixel, ***x****_p_* is the analyzed pixel, and $G_{i}^{\left(t\right)}$ is the specific group.

Then the second step is performed–the average is calculated from the intensity values of the pixels belonging to each group; these calculated estimates become the new cluster centroids according to the formula below:

(10) $$m_{i}^{\left(t+1\right)}=\frac{1}{\left|G_{i}^{\left(t\right)}\right|}\sum_{x_{j}\in G_{i}^{\left(t\right)}}x_{j},$$

where $m_{i}^{\left(t+1\right)}$ is the newly calculated central point of the group, $G_{i}^{\left(t\right)}$ is a specific group, and **x**_j_ is the intensity values of pixels belonging to a specific group $G_{i}^{\left(t\right)}$.

The steps of assigning pixels to groups based on the distance to their centers and recalculating the centers of the groups themselves are performed until the central points of the groups do not change their position or a specified criterion is reached, for example, a predetermined number of iterations is completed, a certain accuracy is achieved, etc.

After determining the exact centers of groups in the form of pixel intensity values, color quantization is performed—all the pixels of the image are replaced by the intensity values of the central pixels of the groups assigned to them. It was chosen to filter the image with the filter described in Section 3.2.1 before processing it with the color quantization method to blur non-informative objects with the background. To execute the algorithm itself, it was decided to create two groups into which pixel intensity values will be divided; the maximum number of iterations is 200, and the maximum accuracy is 0.5. Continuing the analysis of the image, the contours of objects are found, described in Section 3.1.3, and these contours are identified with fixed contours of animal objects; this technique is described in Section 3.1.4.

#### 3.3.2. Results

The image below presents animals and visual representations of the stages involved in the third methodological step—color quantization, followed by thresholding.

**Figure 8:**
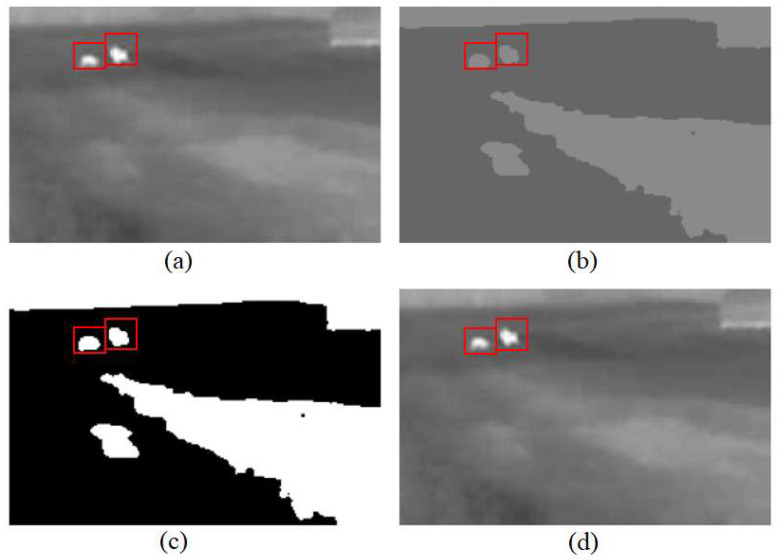
The image processed using a color quantization by the nearest average algorithm. (**a**) The original thermal image captured by the thermal camera. (**b**) The image after applying color quantization, which reduces the number of distinct intensity levels to simplify subsequent segmentation. (**c**) The binary image obtained by applying intensity thresholding to isolate high-temperature regions, corresponding to potential animals. (**d**) The final detected animal contours superimposed on the original image.

During the color quantization process, the decision was made to categorize the colors into two distinct groups. Consequently, as shown in Figure 8b, all color variations in the image are reduced to two shades of gray, differing in lightness. Following the application of thresholding, the lighter gray tones are converted to white, while the darker tones are rendered black. It is important to emphasize that the lighter tones must be mapped to white, as thermal imaging inherently depicts animals using the lightest shades. In the sample image provided, two animal subjects have been identified; in both the original image and those illustrating each methodological stage, the detected animal regions are enclosed within red rectangles.

### 3.4. A Method for Detecting Animals in a Set of Images Based on Motion Detection

Following the analysis of results obtained from processing individual images, a decision was made to implement animal object detection across a sequence of images. In this approach, photographs captured by the camera are analyzed sequentially, enabling the detection of animals through comparative analysis of consecutive frames. The methodology described below focuses specifically on the processing of two successive images.

#### 3.4.1. Method Description

**Figure 9:**
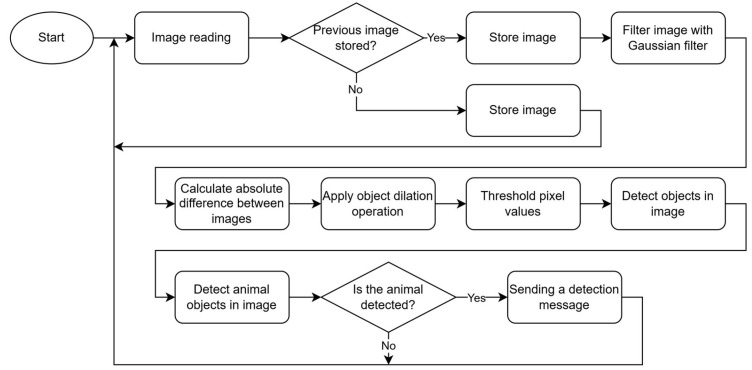
Flowchart of animals’ detection based on motion detection.

A methodology designed to detect animal movement across consecutive frames has been selected for testing. The operational algorithm of this methodology is presented in Figure 9. As illustrated, the process begins with the application of a Gaussian filter to the images. Subsequently, the absolute difference between the preceding and current frame is computed, followed by the application of an object dilation operation to the resulting difference image. The next steps involve thresholding the pixel values, detecting the contours of objects present in the image, and comparing the identified contours with the reference contours corresponding to animal objects.

#### 3.4.2. Absolute Difference Between Images

Each photo obtained from a thermal imager is a picture made up of a certain number of pixels with their intensity values. Therefore, an image can be viewed as a matrix or array of pixel intensities, yet the calculation of the absolute difference between different frames can be performed. This makes it possible to identify areas of the image where pixel intensity values have changed significantly, indicating a new edge of the object or an occupied area, and thus conclude that the object has moved. The absolute difference equation is as follows:

(11) $$P_{differential}\left(i\right)=\left|P_{previous}\left(i\right)-P_{curren}\left(i\right)\right|,$$

where ***P****_differential_*(*i*) is the received differential frame, ***P****_previous_*(*i*) is the previous frame, and ***P****_current_*(*i*) is the current image.

The absolute difference module is needed to capture the change between pixels while keeping the intensity value in the correct range of 0 to 255.

#### 3.4.3. Objects Dilation

The object dilation provides an opportunity to fill the holes in the image and connect the adjacent areas. Small differences after calculating the absolute difference between the frames are magnified in order to make it easier to recognize the contours of the objects during the subsequent steps of the methodology [37]. The contours of objects in the picture are expanded using the formula for expanding objects of the grayscale picture type:

(12) $$(P⨁)(x)={sup}_{y\epsilon E}\left[P\left(y\right)+f(x-y)\right],$$

where *P*, *P*(*x*) and *P*(*y*) are, respectively, the image and the pixel of the image with extended objects and with unmodified objects, *f*(*x* − *y*) is the structural function, and *sup_y__ϵE_* is the supremacy selected from a certain amount or the entire set of pixels when the value for the pixel *y* belonging to the given set *E* is selected.

Selected objects are expanded using the uniform structuring element function *f*:

(13) $$f\left(x\right)=\left\{\begin{array}{c} 0,\;\;\;x\epsilon B\\ -\infty ,\;\;\;else \end{array}\right.,\;\;B\epsilon E,$$

where *f*(*x*) is the pixel of the structural element/kernel, *B* is a certain number of pixels belonging to the reference set *E*, and *E* is all the pixels in the image.

By modeling the kernel as a 5 × 5 matrix of units and choosing to return the maximum intensity value instead of supremacy, the feature expansion formula is simplified:

(14) $$(P⨁)\left(x\right)={sup}_{y\epsilon E}\left[P\left(y\right)+f\left(x-y\right)\right]={max}_{z\epsilon B}\left[P(z)\right],$$

where ***P***(***y***) and ***P***(***z***) are the old pixel intensity value and pixel values covered by the kernel, *f*(***x*** − ***y***) is the function of the structural element, and *max_z__ϵB_* is the maximum pixel values.

The object dilation method works on the principle of a moving window [38]. The kernel is scrolled through the image, and the new value for the pixel in the center of the kernel is determined by selecting the maximum intensity value from the pixels covered by the kernel. After completing these steps, the thresholding algorithm for obtaining a binary image is applied, described in Section 3.1.2; the contours in the object are determined following the principle described in Section 3.1.3, and the algorithm described in Section 3.1.4 is applied, in which contours belonging to animals are recognized.

#### 3.4.4. Results

**Figure 10:**
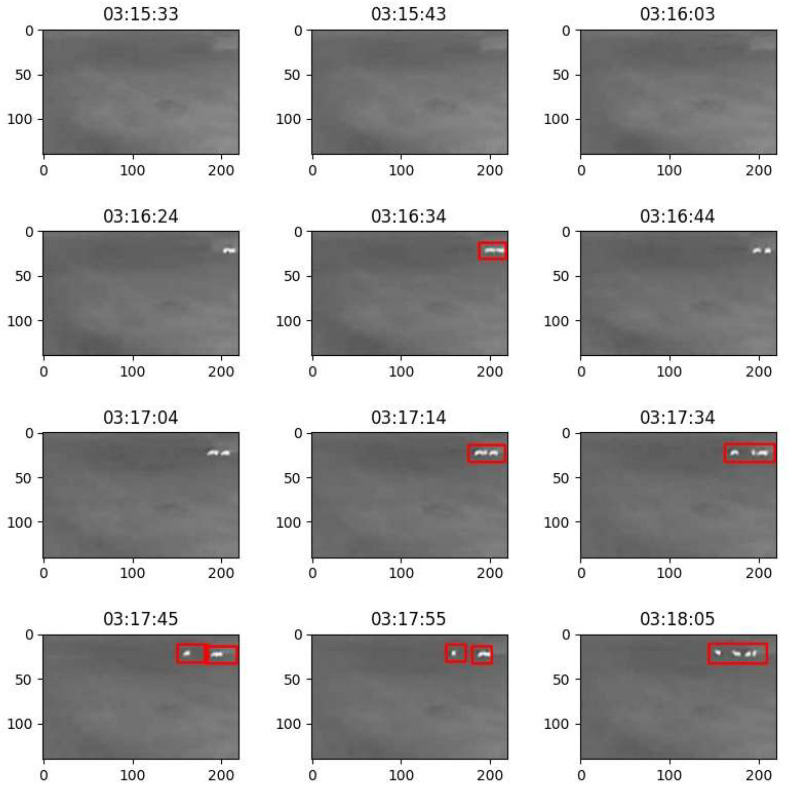
The sequence of the images analyzed using the motion detection methodology.

Figure 10 presents a series of images captured at intervals of 10–20 s and analyzed using the motion detection methodology. In each image, detected and moving animal objects are highlighted with red rectangles. Upon examining the sequence, it is evident that in the second row—specifically, the first and last frames—and in the first frame of the third row, animal outlines are visible but not marked. This occurs even though the bounding rectangles corresponding to the animal objects intersect with the frame boundaries.

This observation indicates that when an animal is not detected in the preceding frame but is detected in the current one—with its contour extending to or intersecting the edge of the image—it can be inferred that the animal has entered the camera’s field of view for the first time. In such cases, the movement of the animal must be assessed based on subsequent frames (as illustrated in the sequence between 03:16:03 and 03:16:24).

When an animal is detected in a preceding frame and continues to be observed in motion, but in the current frame appears at the boundary of the camera’s field of view, it may be concluded that the animal is moving toward the edge of the monitored area. Specifically, in the analyzed sequence (see Figure 10), the animal appears to be moving to the right, away from the potentially hazardous rural road located on the left (as observed between frames 03:16:34 and 03:16:44, and again between 03:16:44 and 03:17:04). However, in these instances, no immediate threat is registered.

### 3.5. A Method for Detecting Animals in a Set of Images Based on the YOLOv8n Neural Network

To provide a comparative benchmark against the classical image processing techniques, a state-of-the-art deep learning methodology was implemented. The YOLOv8n (“nano”) object detection framework was used, as its lightweight architecture is optimized for high-speed inference on resource-constrained embedded systems, such as the target Raspberry Pi platform.

For this study, the processed thermal images after cropping have a resolution of 219 × 134 pixels, covering a field of view that extends up to 427 m, with a key reference point, a building, situated at a distance of 150 m (see Figure 2). The target animals, such as moose (2.0–2.7 m) and roe deer (up to 1.5 m), appear as very small objects in the low-resolution thermal image frames. To quantify this, at a distance of 150 m, a large moose would be approximately 3 pixels long, while a smaller deer or boar would be only 1 to 2 pixels wide. The small size of these objects makes visual recognition challenging and subjective, particularly at greater distances. To mitigate this and ensure accurate data annotation, an expert was engaged to visually verify the presence of animals in the image set.

For training the model, a specialized dataset was prepared, consisting of 809 thermal images captured across different time periods to ensure a diversity of environmental conditions and animal poses. To establish a high-quality ground-truth, an expert manually reviewed the entire image set and annotated the precise location of any visible animals with bounding boxes.

The YOLOv8n model was trained for 40 epochs. Throughout the training, all loss functions (box_loss, cls_loss, dfl_loss) showed a consistent decrease for both the training and validation sets, signaling successful model convergence without significant overfitting. The performance metrics achieved during this phase were highly promising for the model’s final detection capabilities. The mean Average Precision at a 50% IoU threshold (mAP@0.50) rapidly improved and stabilized around a value of 0.95. Furthermore, the more stringent mAP@0.50–0.95 metric steadily rose to approximately 0.7.

To assess the practical performance and generalization capability of the trained YOLOv8n model, an independent hold-out test set was created. This set consisted of 410 previously unseen images, comprising 300 images containing one or more animals and 110 images with no animals present. The images were sampled from various time periods to ensure a robust evaluation.

**Figure 11:**
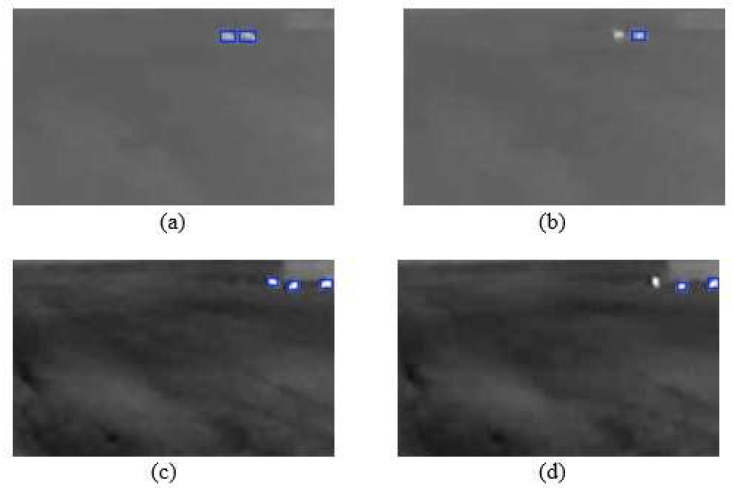
The sequence of images. (**a**) Two animals were detected. (**b**) Only one of two animals was detected. (**c**) Three animals were detected. (**d**) Only two of three animals were detected.

**Figure 12:**
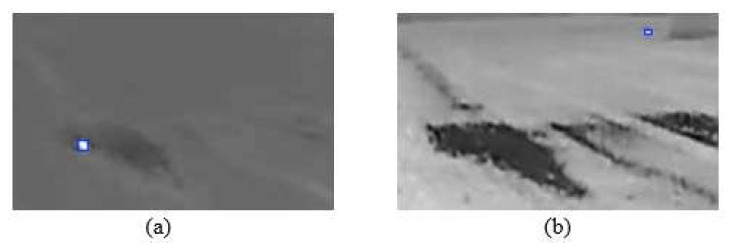
The sequence of images. (**a**) Falsely detected water reflection. (**b**) Falsely detected object.

Figure 11 presents qualitative results of the YOLOv8n model, illustrating both its successes and limitations on images containing animals. Figure 11a,c show instances of correct performance, where the model successfully detected all two and three animals present in the frame, respectively. Conversely, Figure 11b,d highlight cases of missed detections (false negatives). These failures can often be attributed to challenging conditions, such as the significant distance to the animal, the target’s small size, or reduced image clarity due to adverse weather conditions, causing the object’s thermal signature to be too faint for the model to register. Figure 12 illustrates typical scenarios that lead to false positive detections, where the model incorrectly identifies an object in an image with no animals. In Figure 12a, a thermal reflection from a water surface is erroneously flagged as a target. In Figure 12b, the model misidentifies a stationary metallic object located near a building. It is noteworthy that in both of these cases, the source of the false positive is static. Therefore, such errors could potentially be filtered out by supplementing the system with a motion-based algorithm designed to discard detections that remain stationary over consecutive frames.

## 4. Discussion

Wild animals such as deer and wild boars predominantly migrate during nighttime hours, which coincides with the highest incidence of vehicle–animal collisions. Due to the absence of sunlight and other heat-emitting surfaces, thermal imagery captured during nighttime typically contains minimal visible thermal signatures apart from the animals themselves. Environmental conditions such as fog, rain, or snow were not accounted for in the current analysis, as it is challenging to determine the presence or intensity of such weather phenomena solely based on thermal images. These conditions can obscure the thermal contrast and make reliable identification more difficult.

Following the identification of animal objects in the images using various methodologies, a set of parameters was selected to differentiate the methodology implemented within the embedded system:

- The average execution time of the methodology.
- The percentage sensitivity of the methodology.
- The specificity of the methodology as a percentage.
- The accuracy of the methodology as a percentage.

The average execution time of a methodology refers to the duration required by the implemented algorithm to process an input image and produce an output indicating whether an animal has been detected. The algorithm accepts an image as input and returns a binary result regarding the presence of an animal. The equation used to calculate the average execution time is presented below:

(15) $${time}_{average}=\frac{\sum_{i=1}^{N}t}{N},$$

where *N* is the number of tests, and *t* is the duration of one test in seconds.

The sensitivity, specificity, and accuracy of the methodology are statistical measures that, in the context of this study, quantitatively assess the suitability of the methodology for real-world application scenarios.

Sensitivity indicates the method’s ability to correctly identify positive cases—that is, the accurate detection of animal objects in images. The equation used to calculate sensitivity is provided below:

(16) $$sensitivity=\frac{TP}{TP+FN},$$

where *TP* (*True Positives*) is the number of correctly identified instances where an animal is present, and *FN* (*False Negatives*) is the number of instances where an animal is present but not detected by the methodology.

Specificity reflects the ability of the methodology to correctly identify negative cases—that is, to accurately determine when no animal objects are present in an image. In other words, it evaluates the method’s effectiveness in correctly classifying images that do not contain animals. The equation is as follows:

(17) $$specifity=\frac{TN}{TN+FP},$$

where *TN* (*True Negatives*) is the number of correctly identified instances where no animal is present and none is detected, and *FP* (*False Positives*) denotes the number of instances where an animal is incorrectly detected in an image that does not contain one.

Accuracy represents the overall effectiveness of the methodology in correctly classifying both positive and negative cases. It quantifies the proportion of correctly identified instances (both animal presence and absence) among all evaluated cases. The accuracy is calculated using the following formula:

(18) $$accuracy=\frac{TN+TP}{TN+TP+FN+FP}$$

where *TP* (*True Positives*) is the number of cases where animals are present and correctly detected, *TN* (*True Negatives*) is the number of cases where no animals are present and correctly undetected, *FP* (*False Positives*) is the number of cases where animals are incorrectly detected in images without them, and *FN* (*False Negatives*) is the number of cases where animals are present but not detected.

Using the selected evaluation parameters, the described methodologies were tested. A sequence of 89 consecutive frames was selected for the analysis. These frames were manually assessed by an observer to ensure that the chosen sequence included transitions in which animals entered and exited the frame—i.e., some frames contained visible animals, while others did not.

Each selected image was provided as input to the execution algorithm of each methodology. This approach enabled a comparative analysis between methodologies based on single-frame processing and those analyzing pairs of frames—the current and the preceding one.

The following figures present the evaluation results, including metric-based performance indicators and the average execution time of each methodology.

**Figure 13:**
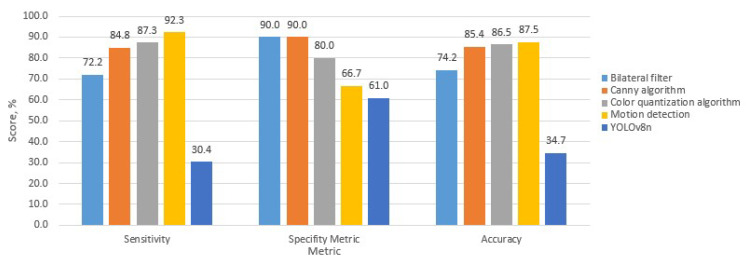
Graphical comparison of the evaluation metrics for the analyzed methodologies.

The graph presenting the evaluation results (Figure 13) of the methodologies according to selected metrics indicates that the highest sensitivity is achieved by the motion-based methodology. The highest specificity is demonstrated by the methodologies employing the bilateral filter and the Canny edge detection algorithm. The greatest overall accuracy is observed in two methodologies: the color quantization-based and the motion-based approaches.

**Figure 14:**
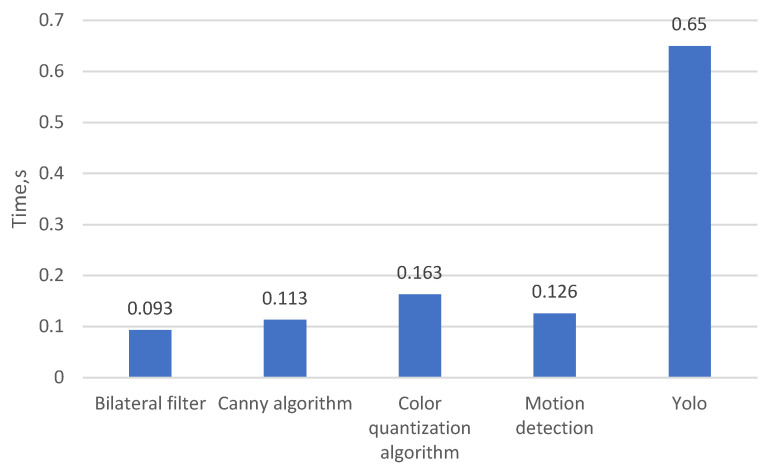
Graphical comparison of the execution time across the evaluated methodologies.

The graph in Figure 14, which presents the evaluation of methodologies based on execution time, shows that the fastest-performing algorithm is the one employing the bilateral filter, whereas the slowest is the color quantization-based methodology.

The following table summarizes the results of the methodology evaluation, providing the exact values of the measured indicators.

Based on the evaluation of the implemented methodologies using the defined parameters, the results presented in Table 1 show that the average execution time of the first four algorithms ranges from 0.093 to 0.163 s. The time difference between the fastest and slowest methodologies is 70 milliseconds. But the last algorithm based on the Yolov8 lite version is 4 times slower in comparison with the first four. The sensitivity values across the methodologies vary between 30% and 92.31%, with the highest sensitivity achieved by the motion-based methodology. To evaluate the performance of the proposed algorithms, all methodologies were implemented in Python 3.8 and executed on the PYNQ-Z2 development board (TUL Corporation, Taipei, Taiwan). The PYNQ-Z2 board was selected due to the integration of the Xilinx Zynq-7000 System-on-Chip (SoC) (AMD Advanced Micro Devices, Santa Clara, CA, USA), which features a dual-core ARM Cortex-A9 processor (AMD Advanced Micro Devices)coupled with a programmable FPGA fabric. This heterogeneous architecture enables software execution on the ARM cores while facilitating hardware acceleration via the FPGA. For experimental consistency, all algorithms were executed on the ARM processor using Python. Multiple test iterations were performed to reduce the impact of transient performance variations, and controlled execution conditions were maintained to minimize interference from background processes.

The highest specificity values, approximately 90%, are observed in the methodologies based on the bilateral filter and the Canny edge detection algorithm, while the Yolov8 methodology demonstrates the lowest specificity, at 61%. In terms of accuracy, the motion-based methodology achieved the highest score—87.50% followed by the color quantization-based methodology, which reached an accuracy of 86.51%. The lowest score was obtained by YOLOv8n.

Considering the obtained results and the specific characteristics of each methodology’s development, the motion-based approach was selected for implementation in the embedded system. This methodology demonstrated the highest sensitivity, indicating superior performance in correctly detecting animals present in the images. Furthermore, it achieved the highest overall accuracy, confirming that, compared to other methodologies, it most reliably identifies animal objects in images.

A key limitation of the current framework is the restricted spatial resolution of the thermal imagery. After preprocessing and cropping, the images cover a broad field of view, with a prominent reference structure used to estimate scale. Target animal species—such as moose, roe deer, and wild boar—appear as extremely small objects within these low-resolution frames. At moderate distances, even large animals occupy only a few pixels, while smaller ones, such as hares, badgers, ferrets, hedgehogs, etc., are represented by minimal pixel clusters.

This limited pixel representation significantly hampers reliable visual detection and introduces subjectivity, especially at extended ranges. To address this challenge and improve annotation accuracy, an expert observer was engaged to manually verify the presence of animals across the dataset, ensuring the integrity of ground truth labels used for algorithmic evaluation.

From a traffic safety perspective, in accidents, smaller animals have less impact and inflict less damage to the vehicle, and therefore, the system detection capability degradation due to limited resolution might be technically mitigated. From a wildlife care perspective, the smaller animal species’ exposure to the road traffic movement affects their migration and population negatively. From a research perspective, future improvements could be achieved by employing higher-resolution thermal cameras, which would enhance object visibility and reduce ambiguity in detection. Additionally, integrating multiple thermal imaging units from different perspectives could expand spatial coverage and enable more robust localization through multi-angle analysis.

## 5. Experiments

**Figure 15:**
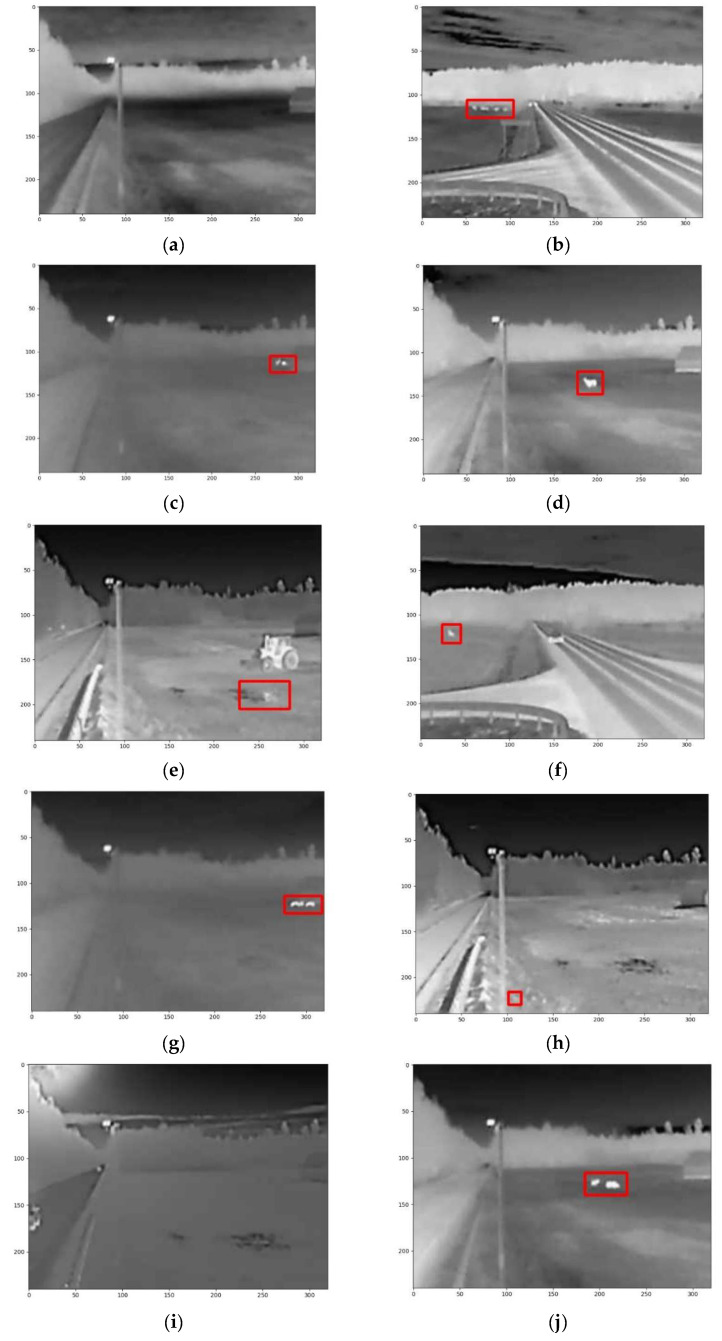
Animal detection in diverse scenarios using motion-based methodology. (**a**) Field without animals, (**b**) group of animals detected on the left, (**c**) group of animals detected on the right, (**d**) one animal detected on the right, (**e**) false detection a puddle was found instead of an animal (**f**) animal detected on the left, (**g**) group of animals detected on the right, (**h**) false detection the small shiny object was found instead of the animal (**i**) field without animals, (**j**) group of the animals on the right.

Following the implementation of the selected methodology within the embedded system and the acceleration of certain processes using the FPGA, an experimental dataset comprising 10 images captured during the different time periods and seasons was assembled (Figure 15a–i). The goal of this analysis was to evaluate the effectiveness of the selected methodology in accurately identifying animal objects across a diverse range of image types.

An analysis of the obtained results (see Figure 15) indicates that, in most cases, the algorithm based on the motion detection methodology successfully identifies animal objects in the images. Neither the season nor the time of day appears to significantly influence the accuracy of the method. This can be attributed to the specific characteristics of the thermal imaging camera, which captures infrared radiation emitted by bodies. As a result, environmental factors such as precipitation, lighting conditions, or other potential interferences do not hinder the algorithm’s performance.

There are also instances in which the algorithm detects and marks animal objects that are either very small or poorly distinguishable (see Figure 15e,h). In such cases, it may be necessary to apply additional image enhancement techniques to increase resolution, allowing for manual verification of the detected object. Alternatively, these images could be classified as false positives if the object’s nature cannot be reliably determined due to image quality constraints.

In summary, in 6 out of the 10 images—taken under varying lighting and seasonal conditions—the animal was correctly identified (see Figure 15b–d,f,g,j). In 2 out of the 10 cases, the algorithm identified a potential animal object, but further processing would be required to confirm the detection (see Figure 15e,h). In the remaining two cases, where no animals were present, the algorithm correctly reported no detection (see Figure 15a,i).

Following the initial experiment, in which the selected methodology was evaluated using ten images depicting different scenarios, four distinct datasets were selected to further assess the methodology’s performance. This subsequent investigation aimed to determine the capability of the motion-based algorithm to distinguish animal objects within sequences of frames obtained from different camera models and exhibiting varying quantities of animal presence. The results of this analysis are presented below.

**Figure 16:**
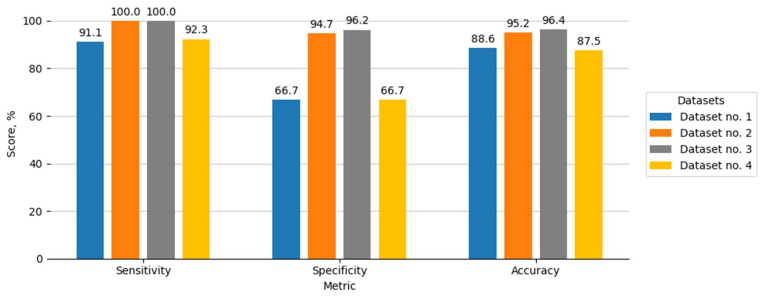
The results of the motion-based detection methodology applied to various datasets representing different camera locations and animal presence scenarios.

As shown in the experimental results (see Figure 16), the evaluation metrics of the methodology vary depending on the composition of the dataset—that is, the total number of images and the number of images containing animal objects.

We systematically assessed each method’s execution time, sensitivity, specificity, and accuracy on sequential thermal frames. The results demonstrate that the motion detection approach yields the highest sensitivity and overall accuracy, critical metrics for reliable wildlife–vehicle collision avoidance, leading to its selection for embedded implementation.

By comparing the total number of images and the number of images containing animals in the datasets (see Table 2) with the corresponding values of the methodology’s performance metrics, it can be concluded that higher metric values are obtained when the methodology is tested on datasets with the largest total number of images but the smallest number of images containing animals. This suggests that the longer an animal remains within the camera’s monitored area, the more likely it is that the motion-based algorithm may produce incorrect evaluations. In such cases, the position of the animal in the frame must change to register motion; otherwise, the algorithm may fail to recognize the animal as a distinct object.

## 6. Conclusions

Following a review of existing methodologies for animal detection and monitoring, it was determined that conventional approaches often incur higher operational costs due to the specificity of the systems employed. Moreover, many of these methods are inherently invasive, either because they require the deployment of machinery or human labor to install equipment at observation sites, or due to the direct attachment of devices to the animals themselves. The analysis also revealed a correlation between the complexity of the sensor system and detection accuracy: specifically, methodologies that utilize a greater number of sensors or those that rely on sensors transmitting more detailed signals tend to achieve higher levels of accuracy in animal identification.

Five distinct methodologies for detecting animal objects in the images were examined. Among them, the motion-based approach demonstrated the highest sensitivity to the specific task requirements, achieving a sensitivity score of approximately 91%. This suggests that, in comparison to the other evaluated methods, the motion-based methodology is the most effective at correctly identifying the presence of animals. Furthermore, this approach also attained an accuracy of approximately 87%, indicating its superior capability to reliably detect animals within images. Based on these findings, the motion-based methodology was selected for implementation in the embedded system.

## Figures and Tables

**Table 1:** Comparison of parameters of different methods for detecting animals.

Methods	Parameters			
	Average Time, s	Sensitivity, %	Specificity, %	Accuracy, %
Bilateral filter	0.093	72.15	90.00	74.15
Canny algorithm	0.113	84.81	90.00	85.39
Color quantization algorithm	0.163	87.34	80.00	86.51
Motion detection	0.126	92.31	66.67	87.50
YOLOv8n	0.650	30.39	61.02	34.68

**Table 2:** Characteristics of image datasets (total number of images and number of images containing animals).

Dataset No.	Dataset 1	Dataset 2	Dataset 3	Dataset 4
Total number of images	89	84	84	17
Images with animals	80	8	4	13

## Data Availability

The data presented in this study are available on request from the corresponding author.
